# Sulfur mass-independent fractionation in subsurface fracture waters indicates a long-standing sulfur cycle in Precambrian rocks

**DOI:** 10.1038/ncomms13252

**Published:** 2016-10-27

**Authors:** L. Li, B. A. Wing, T. H. Bui, J. M. McDermott, G. F. Slater, S. Wei, G. Lacrampe-Couloume, B. Sherwood Lollar

**Affiliations:** 1 Department of Earth Sciences, University of Toronto, Toronto, Ontario, Cananda M5S 3B1; 2 Department of Earth and Atmospheric Sciences, University of Alberta, Edmonton, Alberta, Canada T6G 2E3; 3 Department of Earth and Planetary Sciences and GEOTOP, McGill University, Montreal, Quebec, Canada H3A 0E8; 4 School of Geography and Earth Sciences, McMaster University, Hamilton, Ontario, Canada L8S 4K1

## Abstract

The discovery of hydrogen-rich waters preserved below the Earth's surface in Precambrian rocks worldwide expands our understanding of the habitability of the terrestrial subsurface. Many deep microbial ecosystems in these waters survive by coupling hydrogen oxidation to sulfate reduction. Hydrogen originates from water–rock reactions including serpentinization and radiolytic decomposition of water induced by decay of radioactive elements in the host rocks. The origin of dissolved sulfate, however, remains unknown. Here we report, from anoxic saline fracture waters ∼2.4 km below surface in the Canadian Shield, a sulfur mass-independent fractionation signal in dissolved sulfate. We demonstrate that this sulfate most likely originates from oxidation of sulfide minerals in the Archaean host rocks through the action of dissolved oxidants (for example, HO^·^ and H_2_O_2_) themselves derived from radiolysis of water, thereby providing a coherent long-term mechanism capable of supplying both an essential electron donor (H_2_) and a complementary acceptor (sulfate) for the deep biosphere.

## Abstract (web summary)

Precambrian rocks host a deep hydrosphere, but where dissolved sulfate, crucial for microbial life, comes from is unclear. At 2.4 km depth in the Canadian shield, Li *et al*. find that oxidation of sulfides in the host rocks creates sulfate thus providing a long-term mechanism for the deep biosphere sulfate.

## Graphical abstract

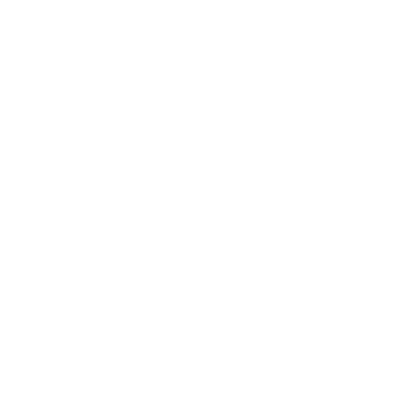

Precambrian crystalline rocks have recently provided evidence for a deep hydrosphere of hitherto unexpected antiquity^1^,^2^,^3^, with noble gas-derived mean residence times for fracture waters of tens of million years (in the Witwatersrand Basin, South Africa^4^,^5^) to billions of years (1.1 to 1.8 Ga at Kidd Creek in the Canadian Shield^3^). At Kidd Creek mine, Timmins, Ontario, the fracture waters from ∼2.4 km depth below the surface were found to contain the most radiogenic helium (He), neon (Ne) and argon (Ar) isotope signatures identified so far in groundwater, while the presence of excess ^129^Xe in the waters was attributed to remnants of the last regional incursion of metamorphic fluids that occurred at ∼2.6 Ga (ref. 3). The discovery of these fracture waters flowing out of boreholes at rates of up to a litre per minute (ref. 3) provides novel insights into the fluid and atmospheric history of the early Earth that are complementary to conventional approaches that have focused exclusively to date on mineral phases and fluid inclusions^6^,^7^.

Deep fracture waters in the Precambrian Canadian Shield rocks are generally characterized by high salinities (total dissolved solids up to 325 g l^−1^), Ca-, Na-, Mg- and Cl-rich compositions, and δ^18^O and δ^2^H values falling well above the Global Meteoric Water Line (GMWL) as a result of extensive fluid–rock interaction^8^,^9^. H_2_ produced by radiolysis^10^ and/or serpentinization can accumulate in fracture waters to millimolar concentration levels, approaching those seen at marine hydrothermal vents^1^. The discovery of chemolithoautotrophic microbial ecosystems coupling H_2_ oxidation to sulfate reduction in the fracture waters at least 2.8 km deep in South Africa's Witwatersrand Basin^5^,^11^ and in deep Fennoscandian groundwater^12^ has generated intense interest in understanding the reactions and energy sources that sustain this subsurface terrestrial biosphere. Although charge balance dictates production of H_2_ via radiolysis must necessarily produce equivalent oxidants^1^,^10^, the important question of the origin of sulfate (the essential electron acceptor to support a sulfate-reducing microbial ecosystem^5^,^11^) has not yet been resolved for these hydrogeologically isolated ancient fracture waters in Precambrian crystalline rocks.

The Kidd Creek Cu-Zn-Ag mine, where the samples in this study were collected, is located in the southwestern Abitibi greenstone belt in the Superior Province of the Canadian Shield (Supplementary Fig. 1), and is currently the deepest base metal mine in North America. The Kidd Creek ore deposits are typical volcanogenic massive sulfide (VMS) deposits^13^ formed from low-temperature (∼250 °C) hydrothermal systems below the seafloor with some mixing of seawater^14^,^15^. The sulfide ores occur within a set of Archaean igneous and (meta-)sedimentary rock sequences (Supplementary Figs 2 and 3). The igneous rocks are ultramafic, mafic (gabbros and basalts) to felsic (mainly rhyolites) in composition^16^,^17^. The (meta-)sedimentary rocks vary from clastic graywackes to graphitic argillites^18^.

The formation of the rhyolites that bounds the main ore bodies was initiated as a result of rifting of a back arc-like basin, similar to the Lau Basin^14^, at 2.717 Ga, reached peak at 2.716 Ga and extended to 2.711 Ga (refs 19, 20, 21), which bracket the ore formation ages to 2.717–2.711 Ga. The onset of convergence and collapse of rift basin induced calc-akaline volcanism as well as deposition of graywackes between 2.700 and 2.695 Ga. Regional metamorphism and deformation to greenschist facies was initiated as folding in 2.690 Ga and reached peak metamorphism at 2.660 Ga with sporadic intrusions of tonalite, granite and granitoid^20^. A discrete hydrothermal event (maximum temperatures estimated at ∼400 °C) occurred at ∼2.640 Ga (refs 20, 22). Localized, lower-temperature hydrothermal alteration could have continued as late as 2.60 Ga (ref. 23), based on the growth of fuchsite in metasomatized ultramafic rocks, and sericite in rhyolite along steep faults. No major hydrothermal events are thought to have occurred in the Kidd Creek area after 2.60 Ga (ref. 20).

The sulfide minerals in the Kidd Creek ores include major layered massive sulfides in the ore body (formed in well-mixed submarine environments), and minor sulfides in turbidites (representing collapsed vent chimneys) and sinters (representing cooled vent effluent) in graphitic argillite layers between the massive sulfide layers (Supplementary Fig. 3)^14^,^15^. The sulfide minerals at Kidd Creek have previously been shown to have sulfur isotope mass-independent fractionation (S-MIF) signals^15^,^24^. The S-MIF signals are commonly identified as non-zero Δ^33^S and Δ^36^S values, which are defined as Δ^3*x*^S=δ^3*x*^S_measured_−1,000 × [(1+δ^34^S_measured_/1,000)^3*xλ*^−1] (in which *x*=3, 6, ^33^*λ*=0.515, ^36^*λ*=1.90, δ^3*x*^S=1,000 × [(^3*x*^S/^32^S)_sample_/(^3*x*^S/^32^S)_standard_−1], and the standard is Vienna Cañon Diablo Troilite, VCDT). Terrestrial S-MIF is interpreted to arise from photochemical reactions in Earth's early anoxic atmosphere^25^,^26^ and commonly seen in crustal rocks formed before 2.4 Ga (refs 27, 28) but thought to disappear from the geological record after ∼2.3 Ga. The massive sulfide ores have consistently negative Δ^33^S values from 0‰ to −0.19‰ VCDT^15^, while sulfides in turbidites and sinters vary more widely with Δ^33^S from −1.50‰ to 1.76‰ VCDT^15^,^24^ (Supplementary Fig. 4).

Here we report multiple sulfur isotope compositions of dissolved sulfate in the saline fracture waters from the Kidd Creek mine that were the focus of the previous noble gas study^3^. The saline fracture waters are intersected by drillholes (300–600 m-long) at ∼2.4 km depth below the surface and naturally flow out of all the boreholes in this study. We observe S-MIF signals in dissolved sulfate in these waters and demonstrate that the S-MIF signature of dissolved sulfate originates from sulfide minerals in the host rocks. The oxidation of sulfide to sulfate is likely induced by oxidants that are produced by radiolytic dissociation of water, which also produces H_2_. Because the water radiolysis is driven by decay of radioactive elements (for example, U, Th, K) in the host rocks, our results indicate that the fracture waters themselves, by interacting with their host rocks in the Precambrian cratons, can provide both an electron acceptor (sulfate) and a complementary electron donor (H_2_) that in turn could support microbial ecosystems in these isolated subsurface fracture systems.

## Results

### Water geochemistry

**Figure 1:**
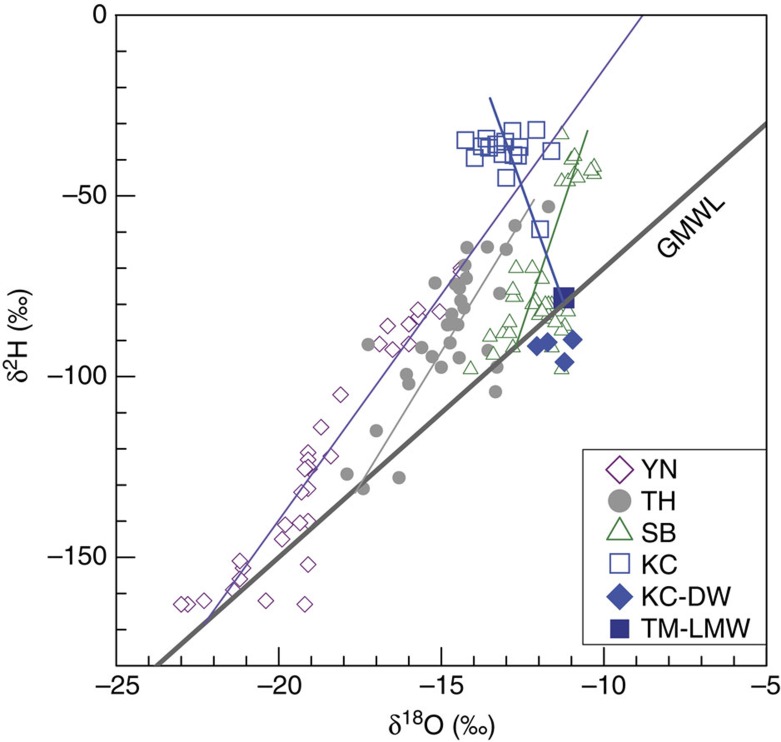
δ^18^O and δ^2^H of subsurface fracture waters. KC represents Kidd Creek (data from this study). Some other sites (YN, Yellowknife; TH, Thompson; SB, Sudbury) in the Canadian Shield (data source: ref. 8), local meteoric water in Timmins, Ontario (TM-LMW), Global Meteoric Water Line (GMWL) and drilling water in the Kidd Creek mine (KC-DW) are also plotted for comparison. Error bars (1*σ*) are smaller than the symbols. Similar to what is observed in the subsurface fracture waters in South Africa^38^,^69^, less saline samples (typically from shallower depths) have oxygen and hydrogen isotope compositions that lie on the GMWL due to mixing with surface water-derived components. While some recharge of surface water to depth cannot be ruled out^38^, the practice of recirculation of surface lake water to depth for use as drilling and service water in the mine is the most likely explanation for the presence of meteoric waters at such depths. KC-DW lies slightly below the GMWL, attributable to water evaporation in the local source lake. Brine fracture waters lie well above the GMWL due to intensive fluid–rock interaction as seen at other sites across the Canada Shield^8^. For each site, a mixing line can be drawn between the most saline fracture water end-member and meteoric water end-members that lie on the GMWL. The samples from this study record some of the most elevated δ^2^H and δ^18^O values ever observed (−31.6‰ to −39.5‰, and −11.6‰ to −14.3‰, respectively) and attest to the lack of significant mixing with surface/drilling waters in the fracture fluids that are the focus of this study.

Fracture water samples were collected (see the ‘Methods' section) over periods from 3 to >60 months after the completion of borehole drilling, with the exception of sample 12262-FW-10.05.07 (marked by ‡ in Table 1), which was collected immediately after drilling finished. The fracture waters flow freely out of the boreholes at rates of up to 770 ml min^−1^, corresponding to a total water discharge of >870 m^3^ or 10^6^ kg from a single borehole over the course of this study. The water samples in this study are among the most saline fracture waters in the Canadian Shield with conductivities mostly around 150 mS cm^−1^ (Table 1) and show strong deviations of δ^18^O and δ^2^H above the GMWL (Fig. 1).

### Dissolved sulfate and multiple sulfur isotopes

Dissolved sulfur species in the fracture waters are dominated by sulfate (97 to 376 μM). Total dissolved sulfide species (H_2_S, HS^−^ and S^2−^) are all below the detection limit of 2 μM. Low sulfide level may arise due to dissolved Fe(II) in the waters (up to 150 μM) scavenging dissolved sulfide through the formation of insoluble iron sulfide minerals.

**Figure 2:**
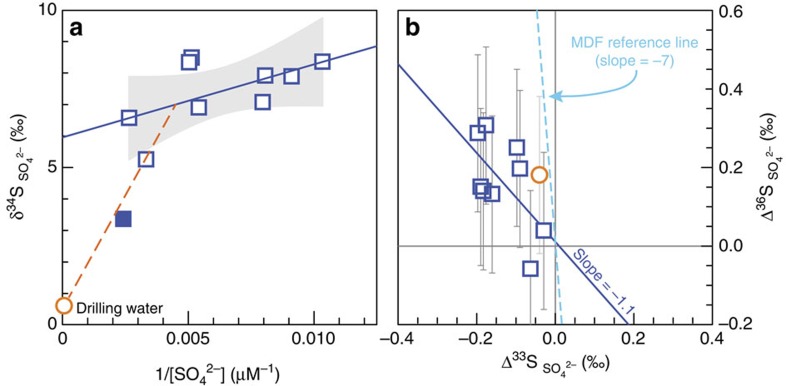
Concentrations and sulfur isotope compositions of dissolved sulfate (open blue squares) in the Kidd Creek fracture waters. Error bars (if not shown, smaller than the symbols) are 1*σ* uncertainty. The data for drilling water (open orange circle) are also plotted in each panel for reference. (**a**) Dissolved sulfate data show a linear trend (solid line). Drilling water sulfate sits far below the linear trend and the 95% confidence interval (shaded area), indicating negligible drilling water contribution to the fracture water samples. The exception is one sample (solid square) that was collected immediately after drilling and hence still shows the effect of mixing with drilling water (also see note ‡ for this sample in Table 1). (**b**) Dissolved sulfate data have a diagnostic MIF Δ^36^S/Δ^33^S slope of −1.1 (solid line), consistent with Archaean sulfur containing minerals, and distinctly different from the classic MDF slope of −7 (dashed line). Note: the sample mixed with drilling water was not included in **b**.

High-precision S-32, 33, 34, 36 measurements (see the ‘Methods' section) of dissolved sulfate revealed the first documentation of S-MIF signatures in groundwaters. Sulfur isotope values for dissolved sulfate in the fracture waters range from −0.03‰ to −0.20‰ for Δ^33^S, −0.1‰ to 0.3‰ for Δ^36^S, and 5.3‰ to 8.5‰ for δ^34^S (Fig. 2). Before this study, similar S-MIF signatures have been exclusively discovered in solid minerals in Archaean host rocks.

Several lines of evidence demonstrate the absence of drilling-related contamination in the fracture water samples. First, δ^18^O and δ^2^H values of the water samples are well above the GMWL, distinct from those of the drilling water (Fig. 1). Second, the drilling water contains significant amount of sulfate (18,320 μM), with isotopic signatures of δ^34^S=0.6‰ and Δ^33^S=−0.04‰ (Fig. 2; Table 1), both very different from those of the fracture waters. In addition, noble gas measurements published previously for the same samples demonstrate the absence of any contamination from modern air or air-saturated drilling water^3^. Only the sample collected immediately after the completion of drilling indicated a small amount of drilling water sulfur contamination (see Table 1 for details).

## Discussion

The Δ^33^S and Δ^36^S values of dissolved sulfate varied from borehole to borehole but remained nearly constant (within analytical uncertainty) for a given borehole over the course of this study. The Δ^33^S values of dissolved sulfate are identical to those of massive sulfide minerals in the Kidd Creek ore deposits (Supplementary Fig. 4). Sulfur isotopic data of dissolved sulfate from all boreholes define a Δ^36^S/Δ^33^S slope of −1.1 with an uncertainty of 2.7 at 95% confidence level. Although the uncertainty is relatively large due to the small size of the measured Δ^36^S values, the estimated slope of −1.1 is close to the value characteristic of sulfides in Archaean rocks (approximately −0.9; ref. 24) but statistically distinct from the canonical mass-dependent fractionation (MDF) slope of approximately −7 (refs 28, 29; Fig. 2b). The presence of sizable S-MIF in ^36^S/^32^S values rules out the magnetic isotope effect associated with thermochemical sulfate reduction by amino acids as the cause^30^. In addition, the non-zero Δ^33^S values measured here (as large as 20 × the external reproducibility) are associated with relatively small positive δ^34^S values (Fig. 2a). These combined characteristics cannot be explained by MDF during either microbial sulfate reduction^28^ or mixing^29^, both of which require substantial changes in δ^34^S to account for comparable changes in Δ^33^S values. Accordingly, we conclude that the observed S-MIF signatures in the fracture water sulfate are of Archaean origin, previously identified only in minerals and rock samples.

Although the fracture waters have been shown to contain remnants of 2.6 Ga hydrothermal fluids based on excess ^129^Xe (ref. 3), our data cannot be explained by preservation of primordial sulfate from the Archaean ocean. A previous study proposed that local Neoarchaean seawater at Kidd Creek had a much more negative Δ^33^S value of −1.5‰ (ref. 15). If such a seawater component were present, it would account for less than 13% of the observed sulfate. Assuming the solid trend line defined by the data in Fig. 2a represents a conservative mixing line with this Neoarchaean seawater end-member, the extrapolated Archaean seawater sulfate component would lie far to the right, at 1/[SO_4_^2−^] values between ∼0.03 to 0.13 μM^−1^, and with a large positive δ^34^S value (∼13 to 36‰). This estimate is inconsistent with δ^34^S value of around 5‰ proposed for Archaean seawater sulfate^31^. Our data also cannot be explained by more recent interpretations of Archaean seawater sulfate characterized by positive Δ^33^S values, based on carbonate-associated sulfate^32^. Finally, the dissolved sulfate in the fracture waters cannot have been sourced from dissolution of Archaean sulfate minerals (for example, barite, gypsum) because such sulfate minerals are not present in the cores we have investigated, nor are they observed in other studies of similar deposits in the area^15^,^33^.

**Figure 3:**
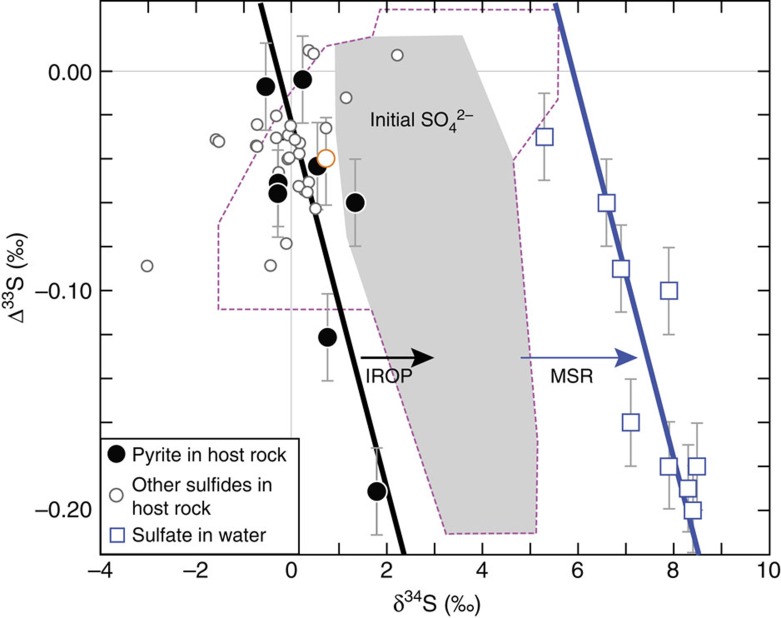
Comparison of δ^34^S and Δ^33^S between dissolved sulfate and host-rock sulfides. Error bars (if not shown, smaller than the symbols) are 1*σ* uncertainty. Dissolved sulfate in drilling water (open orange circle) is also plotted for comparison. The black solid line represents mixing between pyrite minerals with different sulfur isotope compositions^15^ and the blue solid line represents mixing between dissolved sulfate in the fracture waters, respectively. Based on the estimated 1.5‰ to 3.4‰ enrichment^36^ in δ^34^S in the product sulfate during the indirect radiolytic oxidation of pyrite (IROP), the initial isotopic compositions of product sulfate are shown as the area in grey (for pyrite only), or the area defined by the dashed magenta lines (for all sulfides). Significantly, δ^34^S values for dissolved sulfate in the fracture water are instead ∼6‰ more enriched in ^34^S than pyrite, suggesting that an additional process of isotopic enrichment, possibly microbial sulfate reduction (MSR), has affected the measured sulfate (see text).

A simpler interpretation is that the fracture water sulfate originates from the oxidation of sulfide minerals in the Kidd Creek ore deposit. This explanation is supported by the observations that the dissolved sulfate and the host rock sulfides (specifically the massive pyrite) have similar Δ^36^S/Δ^33^S slopes and Δ^33^S ranges (Fig. 3 and Supplementary Fig. 4). As estimated by the overall quantities of major metals (Fe, Cu, Zn, Pb) and sulfur^16^, the massive pyrite accounts for about 70% of the total sulfur budget in the host rocks (Supplementary Table 1), and thus the sulfur isotopic signature of the massive pyrite is representative of the host rock overall. The role of water–rock reaction is also supported by mineralogical observations of dissolution (Supplementary Fig. 5) and re-precipitation (Supplementary Fig. 6) textures on the surfaces of sulfide minerals in drill core samples from the Kidd Creek ore deposit.

The δ^34^S values provide further information to evaluate potential mechanisms of sulfur mobilization from the sulfide minerals into the fracture water reservoir. The fracture water sulfate is ∼6‰ more enriched in ^34^S than the massive pyrite (Fig. 3). Among the possible geological oxidation mechanisms to transform sulfide to sulfate, near surface oxidative weathering of sulfide is common in the oxic atmosphere and hydrosphere after ∼2.4 Ga. Resulting sulfate could have potentially infiltrated into the deep fractures by episodic recharge of surface water. However, oxidative weathering of sulfide involves negligible isotope fractionation^34^ and thus would result in similar δ^34^S values between source sulfide and product sulfate, in contrast to the ∼6‰ offset observed here. Further, δ^18^O and δ^2^H values (lying well above GMWL; Fig. 1), and previously published noble gas measurements^3^ for these same fracture waters, indicate there is no evidence for surface water recharge.

It is therefore more likely that the dissolved sulfate was produced by *in situ* oxidation reactions occurring within the fractures. At the contact between the mineral surfaces and fracture water, such reactions could occur between sulfides (mainly pyrite; Supplementary Table 1) and dissolved oxidants (for example, HO^·^ and/or H_2_O_2_) produced by radiolytic decomposition of water driven by energy released from radiogenic decay of U, Th and K in the host rocks^10^,^35^. This mechanism is hereafter referred as indirect radiolytic oxidation of pyrite (IROP) as it refers to oxidation via radiolysis of water (as opposed to what might be termed the direct mechanism, involving, for instance, γ-rays hitting minerals directly). Although IROP has not been conclusively identified in field studies to date, the process has been shown to produce sulfate in anoxic laboratory experiments on pyrite minerals, and is associated with ^34^S enrichments of 1.5‰ to 3.4‰ from source pyrite to product sulfate^36^. Although these enrichments are generally consistent with the observed ^34^S enrichment in the dissolved sulfate in the fracture waters, the magnitude of ^34^S enrichments due to IROP alone are too small to account for the observed ∼6‰ increase in δ^34^S values of the dissolved sulfate relative to the massive pyrite (Fig. 3).

Further insight can be gained by mass balance modelling of the sulfate concentrations. Using geochemical and experimental data^36^, a rate of sulfate production from IROP in the Kidd Creek rocks can be estimated (see the ‘Methods' section) to a first approximation at 1.7 × 10^−5^ μM per year to 4.9 × 10^−4^ μM per year (Supplementary Table 2). On the basis of the residence times for these waters^3^, the accumulated sulfate concentrations in the fracture waters from IROP would be 1.9 × 10^4^ to 5.4 × 10^5^ μM (for 1.1 billion years) or 4.4 × 10^4^ to 1.3 × 10^6^ μM (for the maximum residence time of 2.6 billion years estimated for these waters). Both estimates are orders of magnitude higher than the measured values of dissolved sulfate (97 to 376 μM), again suggesting that removal processes need to be examined to account for both the elevated δ^34^S values and lowered concentrations of dissolved sulfate observed in the fracture waters.

The fracture waters contain up to millimolar concentrations of dissolved H_2_ and CH_4_, as well as lesser amounts of higher hydrocarbons^37^,^38^, all of which can act as electron donors for sulfate reduction reactions—either thermochemical (TSR) or microbial (MSR). Compared with MSR, TSR is less likely in these systems for two reasons. First, TSR commonly requires environmental temperatures higher than ∼100 °C to proceed^39^. The thermal history of the Kidd Creek terrane since its last low-grade metamorphic hydrothermal event (with peak temperatures of ∼400 °C at 2.6 Ga; ref. 23) is not well constrained. However, studies estimate that the North American Precambrian craton has been stable with essentially no net denudation (estimated rates=0±2.5 m per million years) over at least the last two billion years^40^. Applying this mean estimate to the Kidd Creek area suggests that the Kidd Creek host rocks, in which the boreholes are located at 2.4 km below the surface, have been at their current depth over the past 2.0 Ga. Using a modern thermal gradient of 11.4 °C km^−1^ for the Superior Province^41^ and taking into account the exponetially decayed radiogenic heat along geological time^42^, the temperatures of these fracture waters are estimated to have been as low as 47 °C at 2.0 Ga and 36 °C at 1.1 Ga. Even with the maximum estimate of erosion rate of 2.5 m per million years, the Kidd Creek area would have been eroded by 5 km over the last 2 billion years, or 2.8 km over last 1.1 billion years. This suggests the current sampling site could have had a maximum depth of 7.4 km (corresponding to 143 °C) at 2 Ga, or 5.2 km (corresponding to 77 °C) at 1.1 Ga. These temperature estimates reveal that efficient TSR has been unlikely occurring in the studied fracture waters since at least 1.1 Ga, maybe even since 2.0 Ga. Furthermore, even if we assume TSR did occur, modelling results (see the ‘Methods' section) over temperatures from 50 °C to 100 °C based on open-system kinetic isotope effects (Supplementary Table 3), and on closed-system equilibrium isotope effects (Supplementary Table 4), indicate that the sulfate loss attributable to TSR is at most ∼170 μM, orders of magnitude too small to reduce the predicted sulfate concentrations of 10^4^–10^6^ μM to the measured values of 97 to 376 μM. We turn therefore to an evaluation of MSR to reconcile both the concentration and isotopic patterns identified above.

Two models are explored to quantitatively estimate the possible effects of MSR on a variety of timescales. The first end-member scenario assumes IROP has slowly built up sulfate concentrations to high levels (>1.9 × 10^4^ μM) over the long history of isolation, whereas MSR acted more recently to deplete sulfate to current measured levels, and thus is an episodic scenario. The second end-member scenario explores sulfate production by IROP coupled with simultaneous consumption by MSR, maintaining near modern sulfate levels in a dynamic balance, and thus is a steady-state scenario. Modelling (see the ‘Methods' section) based on the concentration decrease and δ^34^S increase yields estimated instantaneous ^34^S/^32^S fractionation factors up to −1.9‰ for the episodic scenario, and from −1.5‰ to −5.5‰ for the steady-state scenario. Such small magnitudes of sulfur isotope fractionation have not been observed in TSR. Recent studies have identified cases with small fractionation factors for MSR however. Habicht *et al*.^43^ demonstrated fractionation factors with magnitudes of less than −10‰ in cultures grown under sulfate concentrations less than 200 μM, and in natural systems with low sulfate levels. Studies with H_2_ as the substrate, directly relevant to this study, are fewer in number. Hoek *et al*.^44^ were able to produce large fractionation factors (up to almost −40‰) only under low H_2_ growth conditions and slow sulfate reduction rates (SRR). All other experiments conducted with excess H_2_ over a range of sulfate reduction rates, including relatively low SRR (1–5 fmoles per cell per hour) and growth temperatures <70 °C, showed fractionation factors between −1‰ and −6‰, with only a slight increase to a maximum of −10‰ at temperatures between 70 and 80 °C (ref. 44). Similar conditions of excess H_2_ and even slower rates of sulfate reduction are consistent with the ecological scenarios described for the deep microbial ecosystems in saline fracture waters in the Mponeng mine, South Africa^5^,^11^, and are likely applicable as well to the Kidd Creek saline fracture waters. As noted, the thermal history of the Canadian Shield suggests that the temperatures likely remained at less than 50 °C over the past 2 Ga in the Kidd Creek crustal environment. While experimental constraints on sulfur isotope fractionation factors associated with sulfate reduction under conditions of excess H_2_, sulfate limitation, and slow sulfate reduction rates are clearly needed, the experimental demonstration of small fractionation factors in the range of −1‰ to −6‰ (ref. 44) lends support to the microbial sulfate reduction scenarios explored here.

To estimate the catabolic energy available to maintain a sulfate-reducing microbial community at maintenance level in this steady-state scenario, we modelled (see the ‘Methods' section) the hydrogenotrophic sulfate reduction reactions in a manner analogous to that used in modern day high-H_2_ hydrothermal systems^45^ for the measured range in the Kidd Creek fracture waters (6 to 15 mmol l^−1^ H_2_). The potential Gibbs free energy available to H_2_ utilizing sulfate reducers in the fracture waters ranges from −194 to −210 kJ mol^−1^ of sulfate, based on the spectrum of borehole chemistries measured (Table 1). For a steady-state system with an IROP production rate of sulfate between 1.7 × 10^−11^ and 4.9 × 10^−10^ M per year (Supplementary Table 2), this translates to an energy availability between 1.0 × 10^−13^ and 3.3 × 10^−12^ J l^−1^ s^−1^ for hydrogenotrophic sulfate-reducing microorganisms. Given a laboratory-determined maintenance energy of ∼10^−15^ J s^−1^ for a single anaerobic cell at 25 °C (assuming 1 C-mol biomass=24.6 g dry biomass and 10^−13^ g dry cell mass)^46^,^47^, the fracture waters at Kidd Creek could support between approximately 100 and 3,000 cells per litre of fluid, a biomass density consistent with those found in the fracture fluids in South African gold mines^5^,^11^. If the *in situ* energy requirements in these low-energy settings are in fact —two to three orders of magnitude lower than the maintenance energy estimated in laboratory chemostat-based studies^48^,^49^, or similar to basal power requirements estimated in field-based studies of sulfate reducers in anoxic marine sediments (∼10^−19^ to 10^−20^ J s^−1^ per cell^50^), a proportionally larger sulfate-reducing microbial community could be supported.

We have demonstrated that the S-MIF-bearing dissolved sulfate in the saline fracture waters at Kidd Creek originates from sulfides in the Archaean host rocks. The most likely mechanism for sulfate production in these anoxic fracture water systems is the indirect oxidation of sulfide minerals by oxidants from radiolytic decomposition of water, which is induced by the decay of radioactive elements (for example, U, Th and K) in the host rocks. Because both sulfides and radioactive elements have been present since the Archaean, this sulfur-cycling pathway is expected to have occurred over billions of years, with a higher rate earlier in the geologic past due to higher abundance of radioactive elements. This cycling of sulfur provides the mechanism for a long-term source of a major electron acceptor (sulfate) for an important metabolism in the terrestrial deep biosphere. Regeneration of electron acceptors in the presence of reactions that produce electron donors (H_2_, CH_4_) implies that such subsurface ecosystems could exist over long timescales even if isolated from the surface. Finally, the findings have implications for planetary habitability and the exploration for evidence of life on Mars. The mafic and ultramafic lithology of portions of the Martian crust, together with the mineralogical evidence (for example, serpentinization) for long-term water–rock interactions by subsurface groundwaters early in Mars' history^51^, supports the potential for production of electron donors and acceptors that could have sustained microbial life in the Martian subsurface, isolated and protected from the later cold, irradiated and inhospitable surface of Mars^52^.

## Methods

### Sample collection

All the samples were collected from three 300–600 m-long uncased boreholes located at a depth of ∼2.4 km below the surface in a Cu-Ag-Zn mine (Kidd Creek) in Timmins, northern Ontario, Canada. The sampling spanned a time period from 3 to 60 months after drilling completion, providing sufficient time for drilling water to be flushed out of the holes, as attested by δ^18^O and δ^2^H values of water (Fig. 1) and noble gas residence time previously published^3^. For comparison, one fracture water sampled just as drilling finished (indicated by ‡ in Table 1) demonstrates that remaining drilling water contamination can be easily recognized and appears to have affected only this sample to any significant extent. Measured gas flow rates varied from 110 to 11,100 ml min^−1^ and the water flow rates varied from ∼1 to 770 ml min^−1^ (Table 1). Both gas and water flow rates diminished over time, likely as a result of continuous draining of the fracture waters. The sampling techniques for gas have been described in detail in Holland *et al*.^3^. The sampling techniques for waters are summarized below.

Fracture water samples for sulfur isotope analysis were collected by filtering water through a 0.2 micron filter into a 1 liter pre-cleaned Nalgene bottle. Any sulfide species (H_2_S, HS^−^ and S^2−^) in the water were immediately fixed by pre-added extra amounts of CdCl_2_. The samples were taken back to the laboratory in a cooler for further laboratory-based extraction of dissolved sulfide and sulfate. A water sample was first filtered through 0.2 micron filter paper to extract any precipitated CdS and Fe hydroxides. After filtering, the water was acidified to pH=∼2 to remove any dissolved inorganic carbon, which could otherwise interfere with the precipitation of BaSO_4_ in the following step. There was no further precipitation of CdS in any of the samples after acidification. The absence of any such precipitation is indirect evidence for the absence of thiosulfate in these waters, which would otherwise decompose and precipitate yellow CdS. A BaCl_2_ solution was then added with Ba^2+^ in excess of SO_4_^2−^ to quantitatively precipitate dissolved sulfate as solid BaSO_4_. BaSO_4_ was collected by 0.2 micron filtration, dried in an oven, and quantified by mass.

### Sulfur isotope analysis

In the laboratory, sulfide (as CdS) and sulfate (as BaSO_4_) samples were completely converted to H_2_S gas in a customized glass manifold at McGill University by reaction with, respectively, a 1:1 mixture of 6 N HCl and a Cr-reducing solution (for sulfide), and a ‘Thode' reduction solution consisting of 250 ml HI 48%, 410 ml HCl 38% and 121 ml H_3_PO_4_ (for sulfate) at ∼100 °C for 2 h under a stream of pure N_2_ gas. The produced H_2_S was carried by the N_2_ gas flow and trapped on a 4% zinc acetate trap where H_2_S precipitated as ZnS. The latter was further converted to Ag_2_S by adding 0.1 N AgNO_3_ solution. Ag_2_S samples were reacted with excess F_2_ in nickel bombs overnight at ∼250 °C to produce SF_6_. The SF_6_ was purified using a −120 °C cold trap to remove condensable by-products, followed by gas chromatographic separation of non-condensable volatiles. Purified SF_6_ was introduced to a Thermo Finnigan MAT 253 dual-inlet isotope-ratio mass spectrometer at McGill University for ^32^S, ^33^S, ^34^S and ^36^S measurements (at *m*/*z*=127, 128, 129 and 131, respectively). All sulfur isotope data are reported relative to VCDT with analytical reproducibility (1*σ*) better than 0.1‰ for δ^34^S, 0.01‰ for Δ^33^S and 0.2‰ for Δ^36^S. Repeat analyses of the international reference materials IAEA-S-1, IAEA-S-2 and IAEA-S-3 always matched their accepted values within these uncertainties.

### Sulfate productivity

The production of sulfate (in molar; denoted as *P*_(M)Sulfate_) by IROP depends on the absorbed dose rate 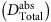 and the sulfate yield per unit of absorbed irradiation energy (*G*):


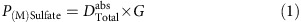


The experimental study^36^ gave a *G* value of 2.1 × 10^−9^ mol m^−2^ Gy^−1^ for sulfate yields by IROP. 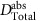 in equation (1) is the total water-absorbed dose rate from decay of ^238^U, ^235^U, ^232^Th and ^40^K in the rock. The decrease in irradiation energy due to transport through the rock to the water-filled fractures is regulated by the stopping power, *S*. Following the study by Lin *et al*.^10^, the 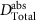 value can be calculated based on the equation below:


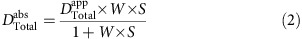


in which *W* is the water/rock weight ratio; and *S* is the stopping power of silicate matrix, which varies from 1.5 for α particles to 1.14 for γ particles^53^. The *W* value is 4.3 × 10^−3^ based on a typical rock density of 2.7 g cm^−3^ and porosity of 1% (refs 1, 3) for crystalline rocks, and measured fluid density of 1.15 g cm^−3^ (*n*=5, 1*σ*=0.01) for our fracture water samples. Porosity in the crystalline rocks (the focus of this study) is entirely dominated by fractures and can be derived from either estimates from the literature for crystalline basement rocks (0.9 to 2.3%; refs 54, 55, 56, 57) or based on fracture porosity function models with depth^58^ that yield similar average values of approximately 1% over 0–10 km depth of the crystalline basement rock^1^. 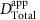 (unit: Gy per year) is the total apparent dose rate due to the decay of ^238^U, ^235^U, ^232^Th and ^40^K, which can be calculated by the following equation^59^:


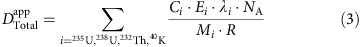


where *C* (unit: parts per million or p.p.m.) is the concentration of the radionuclides in the rocks, calculated based on the concentration of the associated element and the natural abundance of isotopes of that element (that is, U, Th and K). Igneous rocks of the Abitibi Subprovince typically contain 0.91–2 p.p.m. U, 4.31–9 p.p.m. Th and 1.48–2% K (refs 60, 61). *λ* is the decay constant (unit: per year; *λ*=ln[2]/*τ*; *τ* is the half-life). *E* (unit: Mev per decay) is the energy released per decay corrected for neutrino loss in β-particle decay, which is 47.1 MeV for ^235^U, 44.2 MeV for ^238^U, 39.9 MeV for ^232^Th and 0.71 MeV for ^40^K, respectively^62^. *N*_A_ is Avogadro's constant (6.022 × 10^23^ mol^−1^). *M* is atomic mass of corresponding radionuclide (unit: g mol^−1^). *R* is the unit conversion factor from MeV to Gy, and equals to 6.24181 × 10^15^.

The production of sulfate from radiolysis can also be expressed in terms of concentration (denoted as *P*_(*C*)Sulfate_) in the fracture waters by the equation:


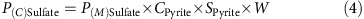


in which *S*_Pyrite_ is the total surface area of pyrite in the rocks. The surface area of pyrite used in this calculation is 226 cm^2^ g^−1^ or 22.6 m^2^ kg^−1^, as used in Lefticariu *et al*.^36^. This parameter is very difficult to constrain for natural deep fracture systems in crystalline rock formations, and thus could introduce a large uncertainty depending on the uncertainty of the actual mineral surface area contacting the water in the fractures. The value does provide a first approximation for the purposes of this study, however (see discussion below). *C*_Pyrite_ is the concentration of pyrite in rocks. Sulfide concentrations vary from ∼5% in average in the non-ore zone (according to four measurements of the core samples from Kidd Creek) to ∼55% on average for the igneous unit containing the massive sulfide ore, which is predominately pyrite, chalcopyrite, sphalerite and galena^13^. Mass balance calculations (Supplementary Table 2) on the elemental inventory data^16^ in the massive sulfide, yield a concentration of pyrite of ∼69 wt% of the total sulfide minerals, corresponding to an estimate of 3.5% pyrite in the non-ore zone and 38% pyrite in the massive sulfide ore zone. All the parameters used for the modelling of sulfate production and calculation results are listed in Supplementary Table 2.

For the purposes of this initial modelling approach, we based our calculation only on the abundance of pyrite, while other sulfides (sphalerite, chalcopyrite, galena) that account for the other ∼30% of the sulfide minerals on which indirect radiolytic oxidation could also act have been neglected in the above first approximation calculations. In addition, modern concentrations of ^235, 238^U, ^232^Th and ^40^K were used in the calculation. Owing to radioactive decay over these timescales, concentrations of these elements would have been higher in the past, particularly in the Archaean. Moreover, the experiments determining *G* values of IROP-derived sulfate were performed under high-energy radiation and the results show that sulfate yields are proportional to logarithmic radiation dose^36^. Thus the *G* value (unit sulfate yield) under the low-dose condition, such as the crystalline rocks in the Canadian Shield as calculated above, is much higher than the value of 2.1 × 10^−9^ mol m^−2^ Gy^−1^ deduced from radiolysis under doses orders of magnitude higher than in the natural environment. Thus, our estimation here is conservative and the actual sulfate production from indirect radiolytic oxidation of sulfide may be higher.

### TSR modelling

If TSR reaction occurs as an ‘open system', where the product sulfide is immediately removed from the site of reaction, the isotopic composition of remaining sulfate as a function of the degree of sulfate reduction should follow the equation below^63^:


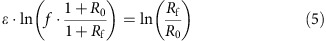


where *ɛ* refers to a kinetic isotope fractionation factor in ‰ between the product sulfide and substrate sulfate, *f* refers to the fraction of remaining sulfate relative to the initial sulfate, *R*_0_ and *R*_f_ refer to the ^34^S/^32^S ratios of initial and final sulfate, respectively. According to the definition of the delta value: *δ*=*R*/*R*_VCDT_−1, equation (5) can be further written as:


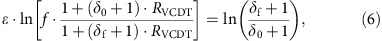


where *R*_VCDT_ equals to 0.04416 (ref. 64), from which the extent of TSR can be calculated as:


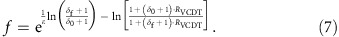


If TSR occurs as a ‘closed system' and equilibrium isotope fractionation is associated with the TSR reaction, the δ^34^S value of remaining sulfate should follow the equation below^63^:


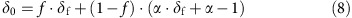


where *α* refers to the equilibrium isotope fractionation factor between sulfide and sulfate. Here, the extent of TSR can be calculated from:


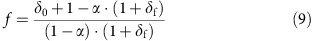


Note that equations (7 and 9) differ from the commonly used Rayleigh distillation equation and the batch model equation, in that equations (7 and 9) use no approximations and thus are more accurate for calculations involving large isotope effects or low *f* values^63^. As the case being tested here involves investigation of systems where *f* approaches very low values (high extent of reaction), these are the most appropriate equations to use.

On the basis of the hydrothermal history discussed in the main text for Kidd Creek, 50 °C and a maximum temperature of 100 °C are used in the calculations for the kinetic isotope fractionation model, and the equilibrium isotope fractionation model for TSR.

### MSR modelling

In the episodic scenario of MSR, ^34^S enrichment factor can be calculated by equation (5) assuming initial sulfate concentrations from IROP as 1.9 × 10^4^ to 1.3 × 10^6^ μM (Supplementary Table 2) and the remaining sulfate concentrations as the observed values of 97 to 376 μM. In the steady-state scenario, the input (IROP produced sulfate) balances output (MSR consumed sulfate) and thus *δ*^34^S value of the produced sulfide must equal that of the supplied sulfate. The standing stock of dissolved sulfate in the water at steady state must then differ from these two by the net fractionation factor (*ɛ*; ref. 65), expressed as:


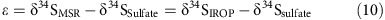


### Free energy and cell density support by sulfate reduction

To conceptualize the catabolic energy available to maintain a sulfate-reducing microbial community in the fracture waters, we applied a thermodynamic modelling approach similar to that used in modern day high-H_2_ hydrothermal chemolithotrophic ecosystems^45^. The Gibbs free energy of the hydrogenotrophic sulfate reduction reaction (Δ*G*_r_):


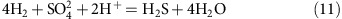


was calculated according to:


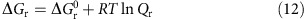


where Δ*G*^0^_r_ is the standard Gibbs free energy of reaction, *R* is the ideal gas constant, *T* is the temperature and *Q*_r_ is the reaction quotient, which accounts for the chemical composition of the mixture and is given by:


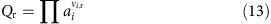


where *a*_*i*_ is the activity of the species *i* raised to its stoichiometric reaction coefficient, *ν*_*i,*r_.

The standard Gibbs free energy of reaction (Δ*G*^0^_r_) was calculated with the Helgeson–Kirkham–Flowers equations of state^66^,^67^ at 25 °C and 1 bar. The aqueous activities of sulfate and water were calculated at 25 °C and 1 bar using Geochemists Workbench 10.0 and the standard thermo_hmw.tdat thermodynamic database^68^, which uses the Pitzer equation-based Harvie–Møller–Weare activity model. The Pitzer approach was chosen rather than Debye–Hückel owing to the high ionic strength of the fracture waters (up to several millimolars). These calculations were performed at 1 bar owing to a lack of pressure-dependent Pitzer coefficients in the literature; however, at estimated *in situ* lithostatic pressures of ∼500 bar, the change in dissolved species distribution would not impart significant effects on these calculations. The molality of measured H_2_ (6.0 to 15 mM) was based on measured gas-to-water flow ratios, the ideal gas law and H_2_ solubility at the inferred 500 bar pressure. Sulfide species are below detection (<2 μM) in the fracture waters, thus a reasonable range in H_2_S molality of 10^−8^ to 10^−6^ M was estimated, assuming equilibration with the pyrite–pyrrhotite–magnetite buffer at 25 °C and 1 bar. Activity coefficients for these uncharged dissolved volatile species were assumed to be unity.

### Data availability

All data supporting the reported findings can be found in the Supplementary Information File.

## Additional information

**How to cite this article:** Li, L. *et al*. Sulfur mass-indepedent fractionation in subsurface fracture waters indicates a long-standing sulfur cycle in Precambrian rocks. *Nat. Commun.* **7,** 13252 doi: 10.1038/ncomms13252 (2016).

**Publisher's note:** Springer Nature remains neutral with regard to jurisdictional claims in published maps and institutional affiliations.

## Supplementary Material

Supplementary Information Supplementary Figures 1-6, Supplementary Tables 1-4 and Supplementary References

## Figures and Tables

**Table 1:**
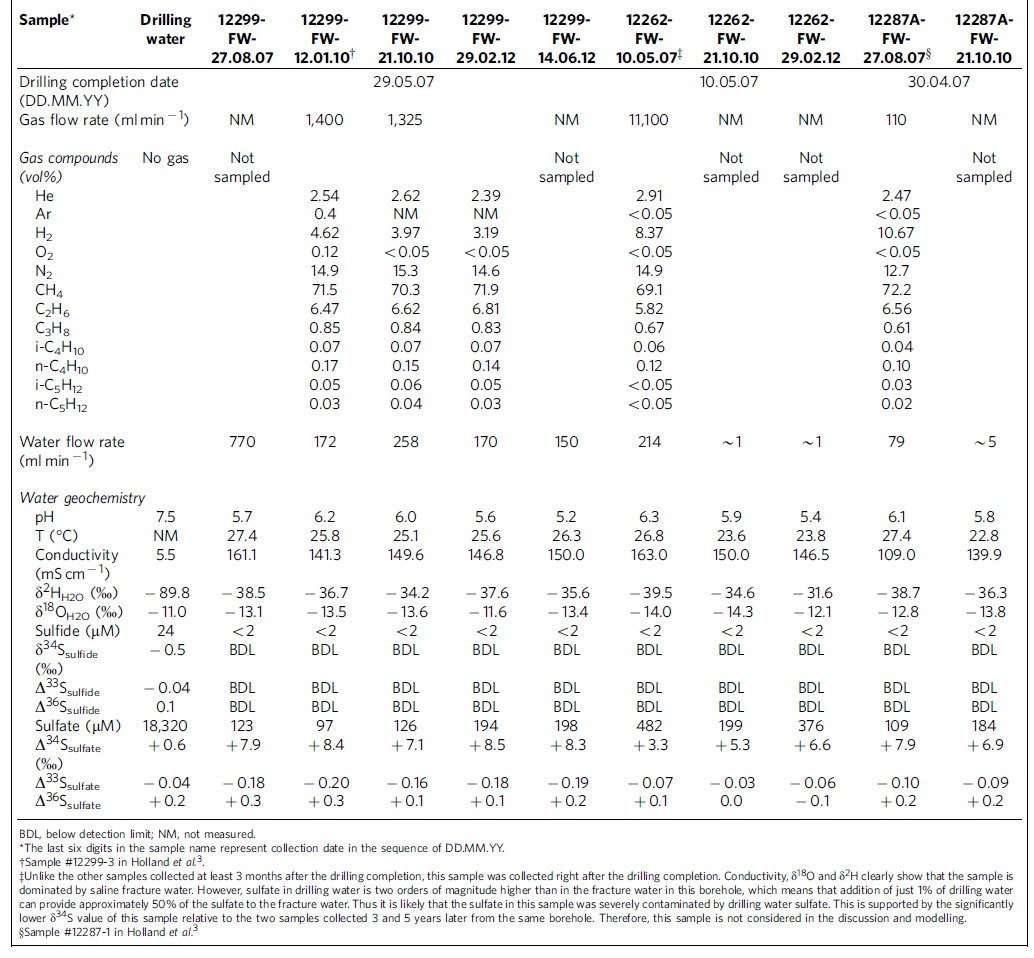
Geochemical and isotopic characterization of fracture waters and dissolved sulfur species.
