# Supplementary material for: Sulfur mass-independent fractionation in subsurface fracture waters indicates a long-standing sulfur cycle in Precambrian rocks
Source: Nat Commun. 2016 Oct 27;7:13252. doi: 10.1038/ncomms13252 (PMC5095282; doi:10.1038/ncomms13252)
Supplement: Supplementary Information — Supplementary Figures 1-6, Supplementary Tables 1-4 and Supplementary References [file ncomms13252-s1.pdf]

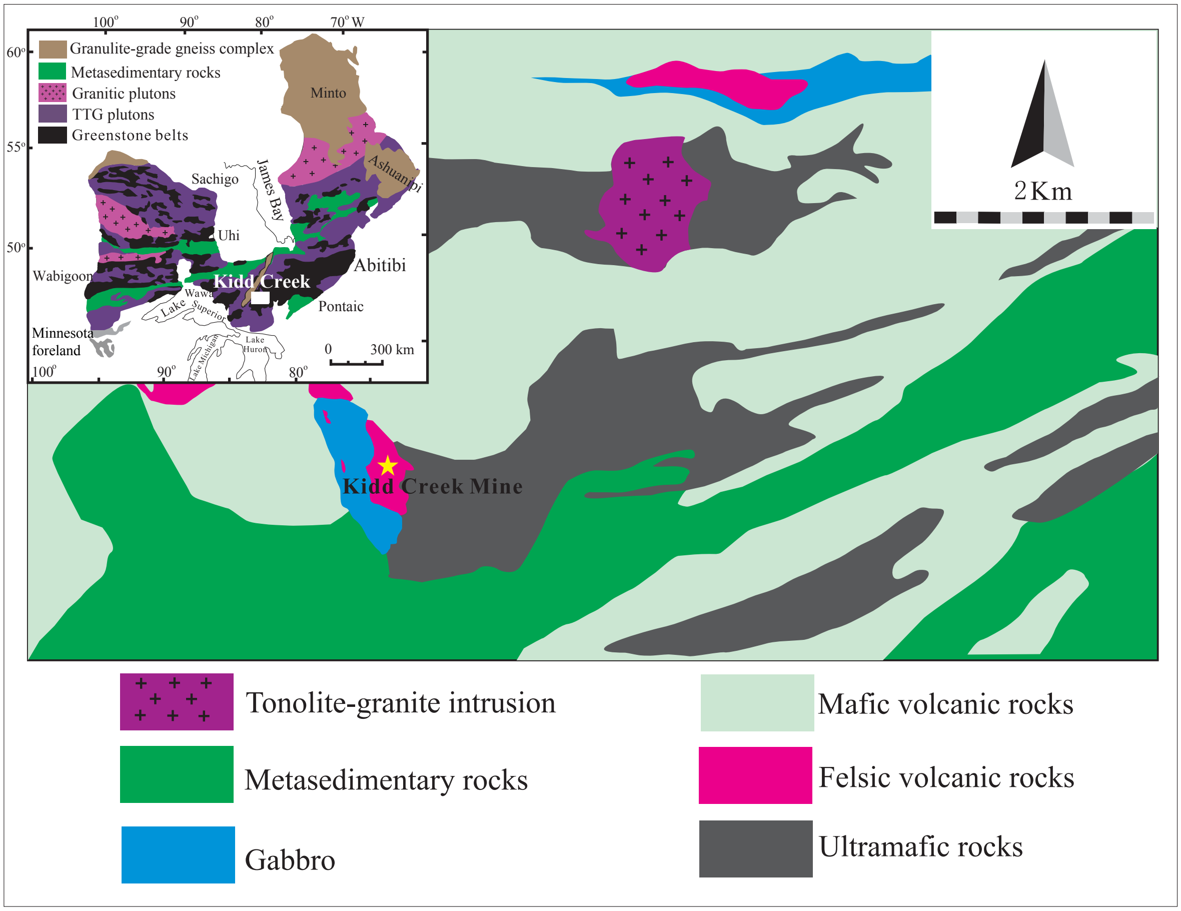

Supplementary Figure 1: Geological maps of the Abitibi Greenstone Belt, Superior Province of the Canadian Shield (inserted panel; modified from Hoffman[1])**, and the geoloic setting for the Kidd Creek mine, Timmins, Ontario** (modified from Barrie[2])**.**

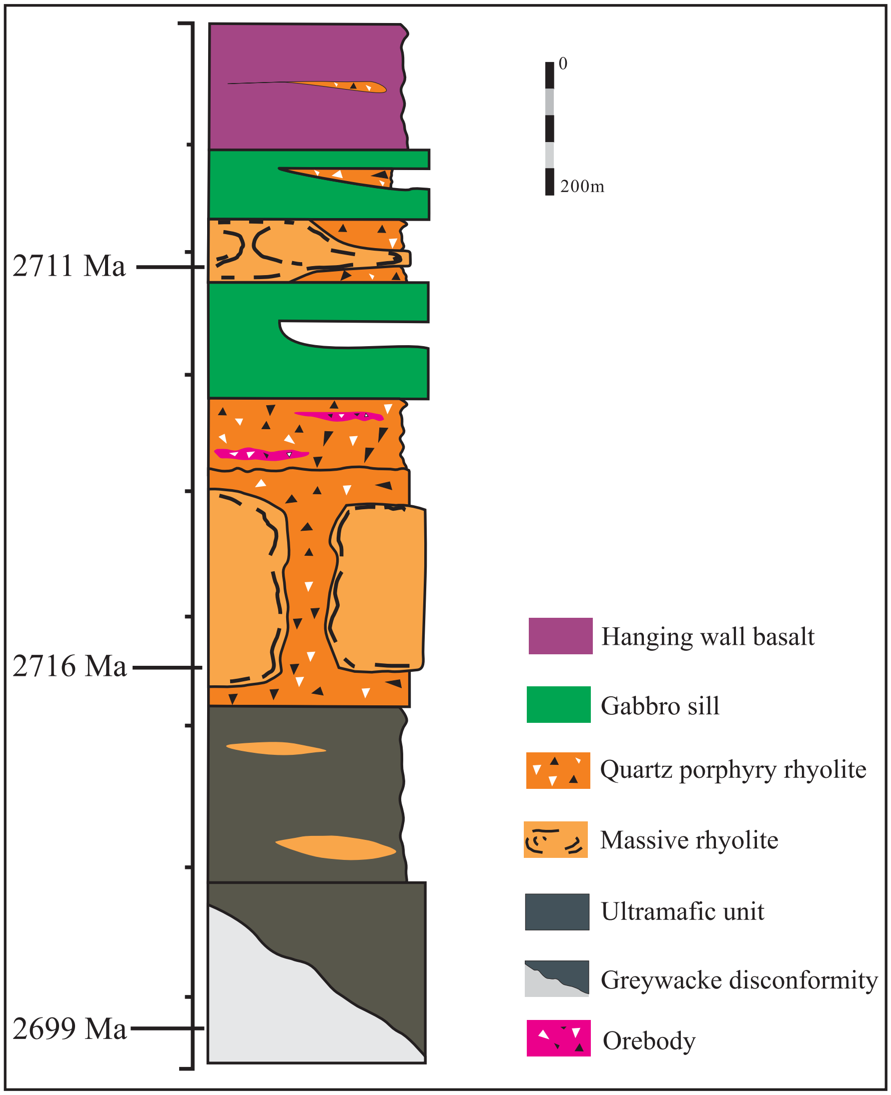

Supplementary Figure 2: Simplified stratigraphic column of the rocks bounding the ore deposits at Kidd Creek (modified from Prior et al.[3]). Formations are shown in original approximately horizontal depositional orientation. Deposits laid down originally approximately horizontally are now steeply dipping sub-vertical units, with boreholes drilled approximately perpendicular to the formation boundaries and intersecting the major units.

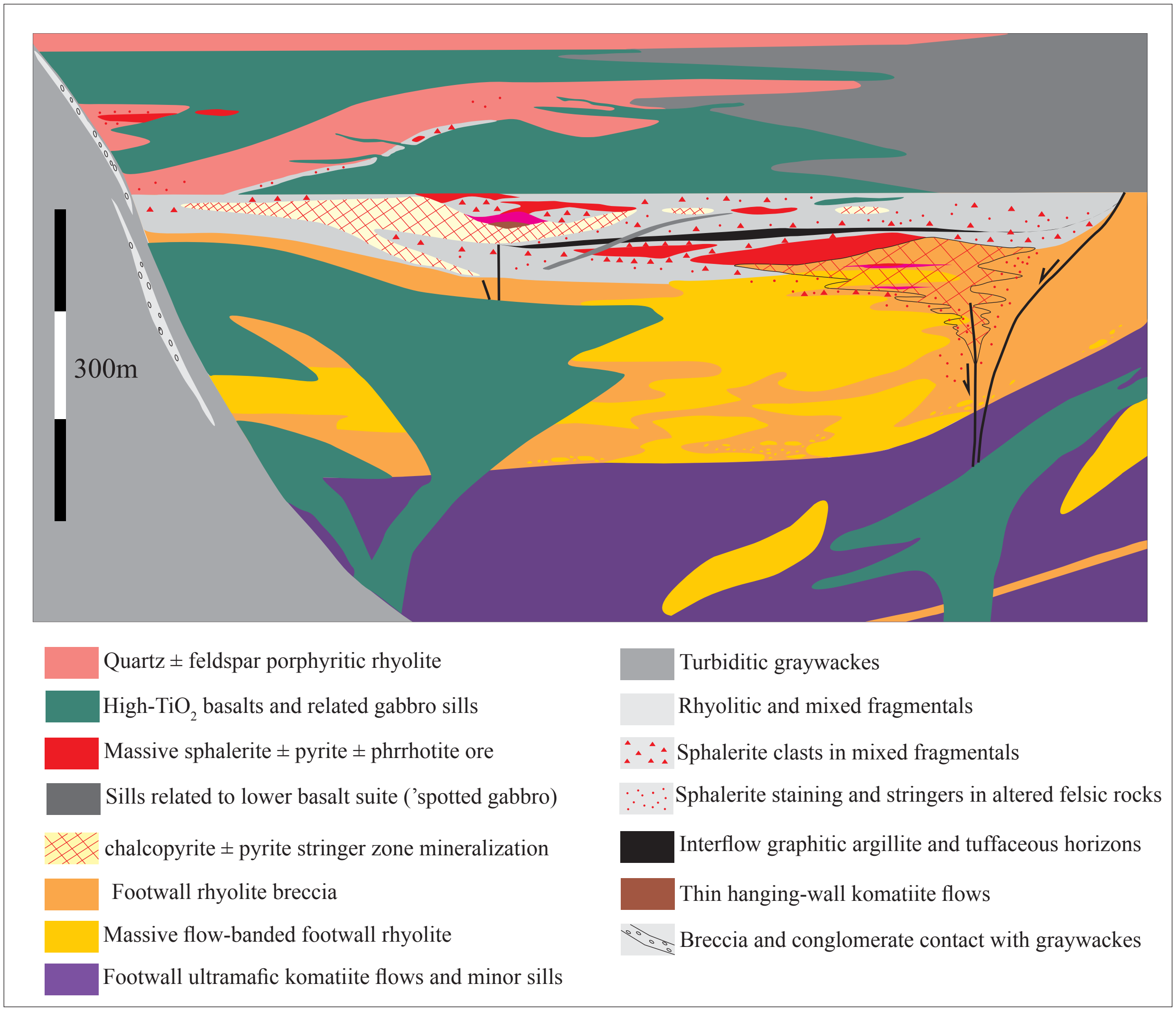

Supplementary Figure 3: Simplified stratigraphy of Kidd Creek geologic setting. Massive hydrothermal deposits and other units are shown in original approximately horizontal depositional orientation (after Bleeker et al.[4]). Deposits laid down originally approximately horizontally are now steeply dipping sub-vertical units.

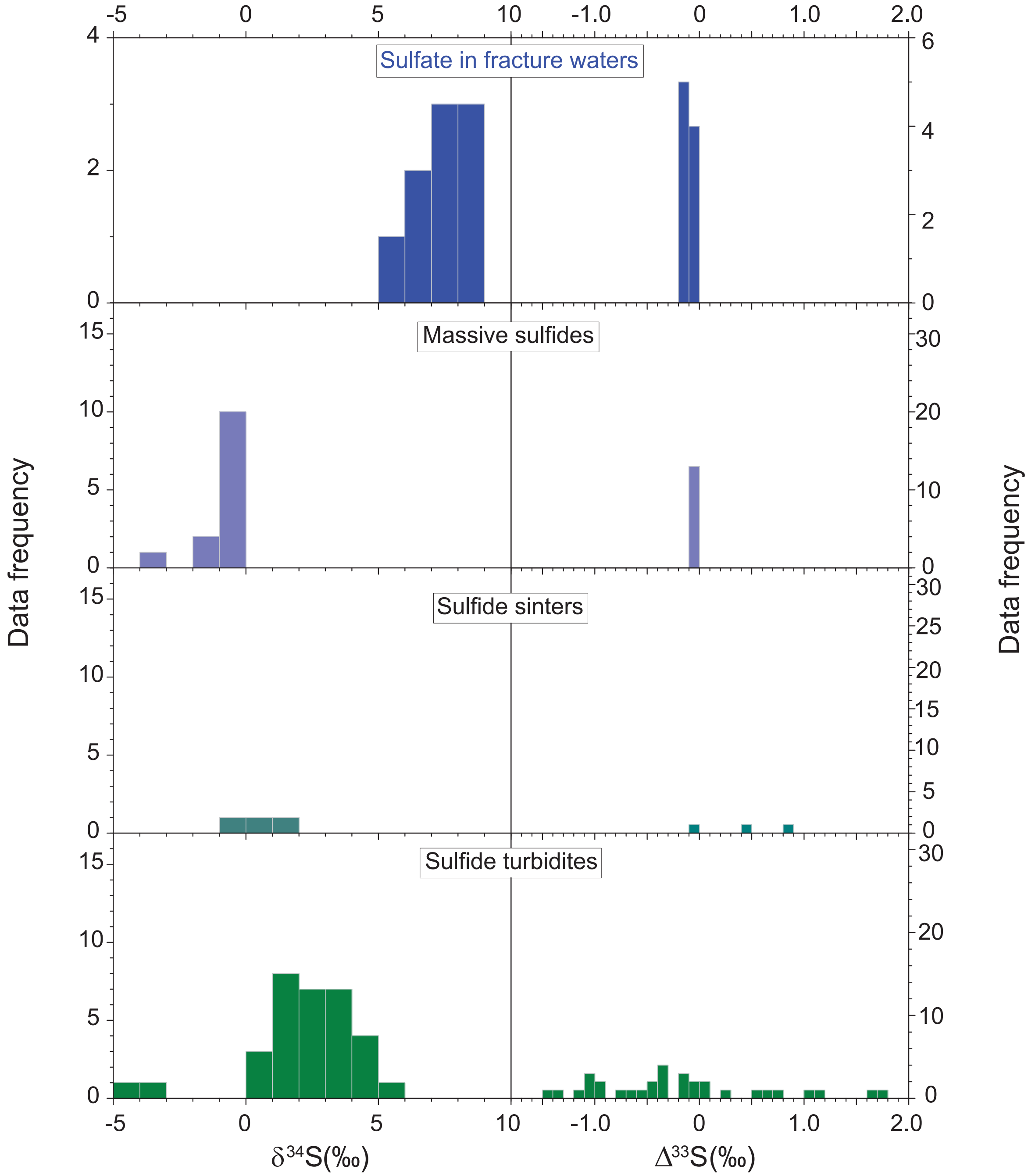

Supplementary Figure 4: Distribution patterns of $\delta^{34}S$ and $\Delta^{33}S$ for dissolved sulfate in fracture waters and sulfide minerals in the Kidd Creek ore deposit. Most data are from the massive sulfide ores (n = 36; second panel)[5]. Fewer data are available for sulfide sinters (n = 3)[5] and sulfide turbidites (n = 32)[5,6] (bottom two panels) which overlap the $\Delta^{33}S$ values of the fracture waters and massive sulfides but also show a wider range of values. The mineralization and associated sulfur budget at Kidd Creek is dominated by the massive sulfide ore bodies[7] and hence these values provide the most representative range for the host rocks.

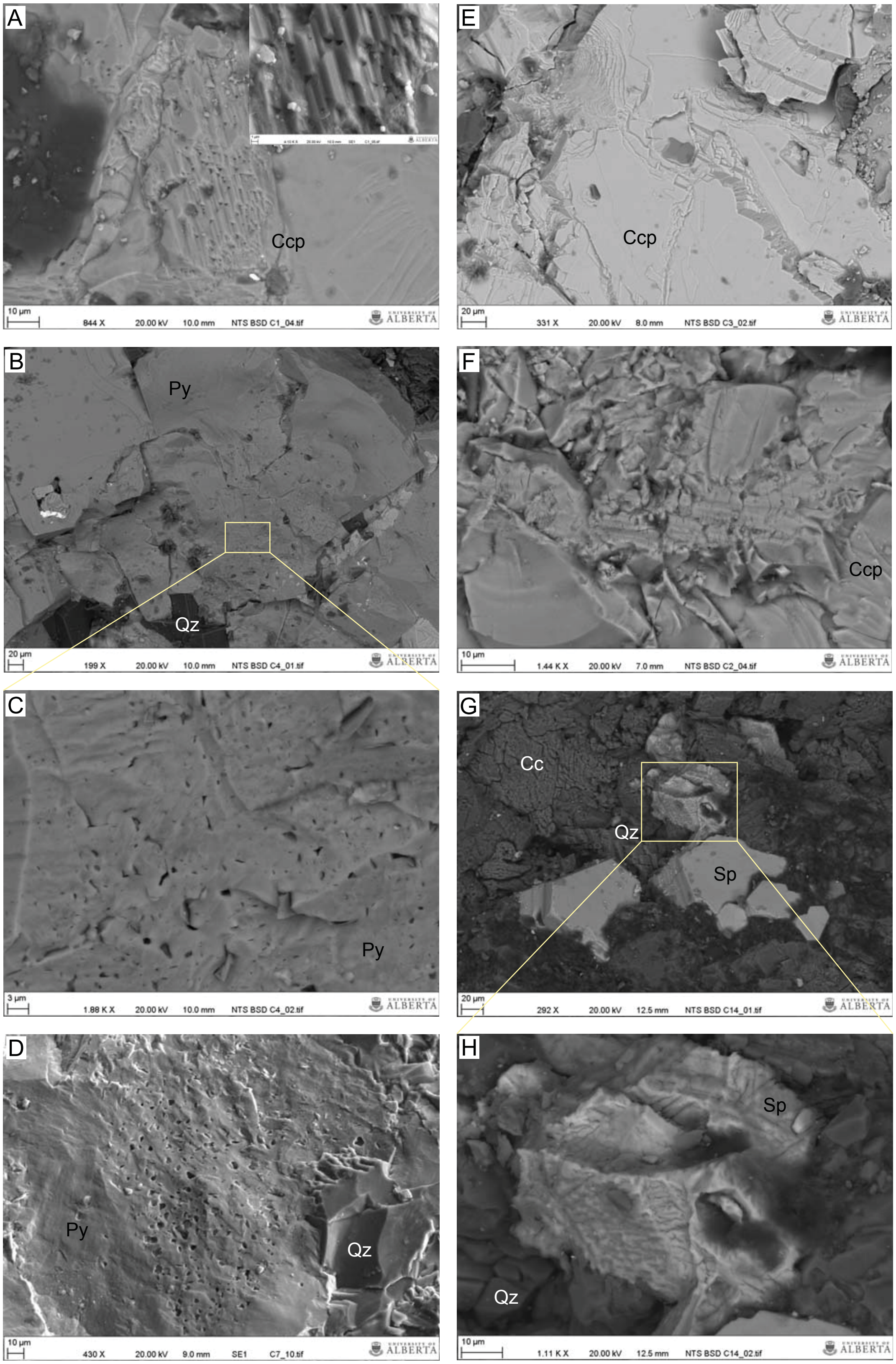

Supplementary Figure 5: SEM images of sulfide minerals in the Kidd Creek ore samples show various surface morphological features. The mullions and trigonal cavities in panel A

and sharp edges in panel E are likely caused by brittle breakage during either drilling and/or sample preparation for SEM analysis. In contrast, panels B-D (C is an inset of B) show clear evidence of dissolution pits, and the smooth channels on panels F-H (H is an inset of G) are interpreted as etching channels resulting from water-rock interaction. Panels B-D show micro-cavities with various shapes and sizes on the surfaces of two pyrite grains. The random distribution of these cavities, and their heterogeneous shapes and sizes are inconsistent with fluid inclusions, which typically exhibit a linear or zoned distribution pattern. The cavities are instead very similar to dissolution pits observed in the laboratory experiments of acidic dissolution of pyrite[8]. Panel F shows complex dissolution features on the surface of a chalcopyrite grain that may be associated with etching products by the contacting water. Several stripes at scales of >10 µm extend in different directions and are always associated with abundant (sub)perpendicular distributary lineae (a few to sub µm scale). Some of the stripes have been truncated by larger, more irregular channels. The small lineations may represent a relatively low degree of etching whereas the irregular channels, which are likely produced by coalescent etch pits[8], represent higher degrees of etching. Panel G shows a few sphalerite grains in a carbonate-rich domain. The grains on the lower half of the image have pits of varying sizes on their top surfaces. Possible reaction cortexes are also seen on all the grains, one of which is enlarged in panel H. The surface of the grain on H clearly shows pronounced roughening and depassivation with crisscross channels resembling etched valleys on the sulfide cortex. The dissolution and etching features described above are commonly seen on the surface of sulfide minerals in rocks from the Kidd Creek mine. Mineral abbreviation: Cc = calcite; Ccp = chalcopyrite; Qz = quartz; Py = pyrite; Sp = sphalerite.

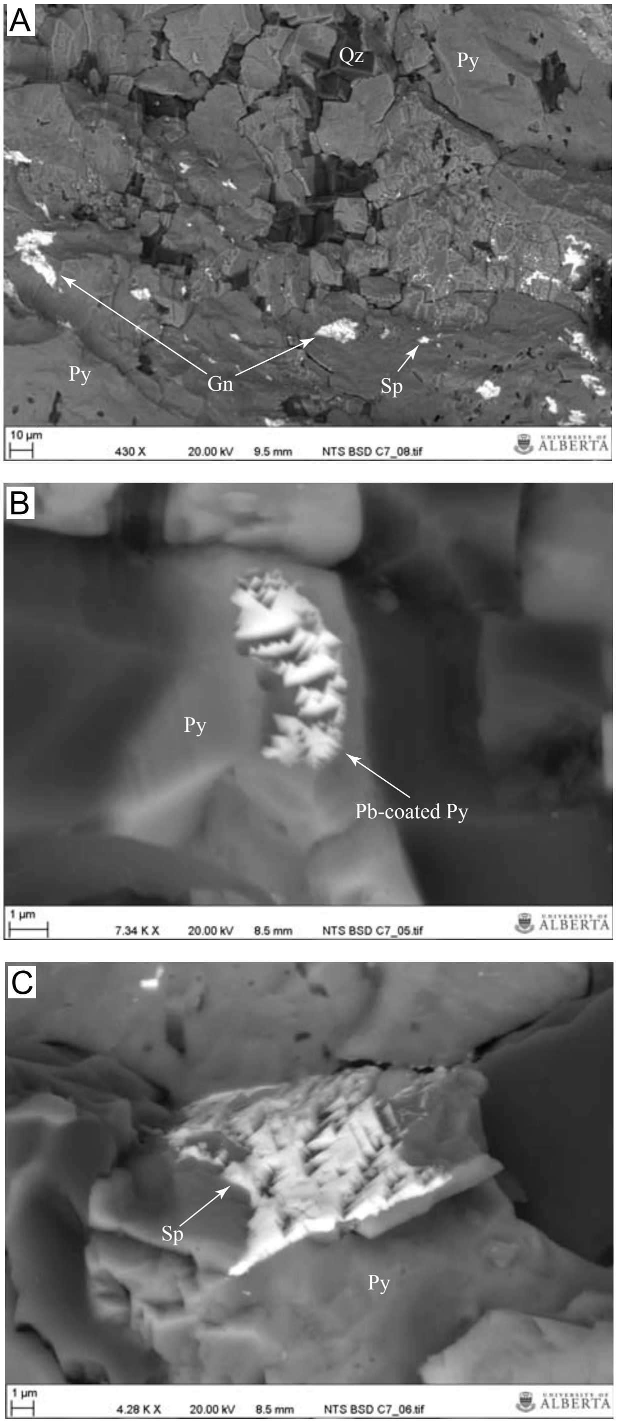

Supplementary Figure 6: SEM images show multiple episodes of re-precipitation of secondary sulfide minerals in primary pyrite mineral grains in the host rocks. (A) Anhedral secondary sulfide minerals, mainly galena (Gn) and sphalerite (Sp), both shown as high brightness in this image because of the high mean atomic mass of PbS and ZnS, completely filled the relatively large angular void spaces (a few to tens of μm) in a primary pyrite (Py) grain (grey area). The characteristics of the void spaces that host Gn and Sp are distinct from dissolution pits, which are much smaller and smoother (Supplementary Figure 5). The

dissolution pits and etching lineations are widespread on Py surfaces, as well as on the galena and sphalerite fillings. These features indicate that Gn and Sp filling the void spaces were deposited at a relatively early stage, though it is not possible to determine whether they were precipitated in high-temperature hydrothermal fluid or subsequently in lower temperature fracture water. In contrast, some other sulfide fillings, i.e., Pb-coated Py (bright grains) in panel B and "seamount chains" made of Sp (bright grains) in panel C, display a different style of precipitation. Their common features include (1) grains that are mainly composed of small (sub-micron) euhedral octahedral crystals, (2) without any obvious dissolution features that are instead observed on the surface of surrounding pyrites, and (3) incomplete filling of void spaces. These features suggest at least two conditions. Firstly, secondary sulfide minerals may have precipitated relatively recently and have not yet been influenced much by dissolution. Secondly, the growth rate of the secondary sulfide minerals has been very low, suggesting a dissolved sulfide-poor environment. This latter point suggests a lower temperature system — a high-temperature fluid would likely assimilate a significant amount of sulfide from water-rock reaction. The low-temperature and sulfide-poor conditions are consistent with our model suggesting extremely slow reaction rates of indirect radiolytic oxidation of sulfides and subsequent sulfate reduction during water-rock interaction.

Supplementary Table 1: **Mass balance calculation of pyrite concentration in massive sulfide ores.**

Mineral	Mineral formula	Metal	Metal quantity (Mt)	Metal molar mass (g mol^{-1})	Metal quantity (×10^9 mol)	S Equivalent quantity (mole)	Mineral equivalent quantity (mol)	Mineral molar mass	Mineral weight (×10^{12} gram)	Mineral wt%
Sphalerite	ZnS	Zn	8.4¶	65.38	128.48	128.48	128.48	97.44	12.52	17.9
Chalcopyrite	$CuFeS_2$	Cu	3.1¶	63.546	48.78	97.57	48.78	183.51	8.95	12.8
Galena	PbS	Pb	0.3¶	207.2	1.45	1.45	1.45	239.26	0.35	0.5
		Total S	33¶	32.06						
Pyrite	FeS_2		Unknown		Unknown	801.83	400.91	119.965	48.10	68.8

¶ Data from Hannington et al.[9]

Supplementary Table 2: Calculation of sulfate production by indirect radiolytic oxidation of pyrite (IROP).

	^{238}U	^{235}U	^{232}Th	^{40}K	*Pyrite concentration*	
					3.5%	38%
Decay energy (Mev)	44.2	47.1	39.9	0.71		
Concentration (ppm)†	0.90-1.99	0.0066-0.014	4.31-9	1.73-2.34		
Half-life τ *(yr)*	4.468E+9	7.038E+8	1.405E+10	1.248E+9		
Atomic mass ($g\ mol^{-1}$)	238.05	235.04	232.04	39.96		
D^{app} ($10^{-3}\ Gy{\cdot}yr^{-1}$)	2.51-5.52	0.125-0.275	3.53-7.37	1.65-2.23		
D^{app}_{Total} ($10^{-3}\ Gy\ yr^{-1}$)			7.81-15.40			
D^{abs}_{Total} ($10^{-5} Gy\ yr^{-1}$) ↕			3.81-9.87			
$P_{(M)Sulfate}$ ($10^{-14}\ mol\ m^{-2}\ yr^{-1}$)			8.01-20.72			
$P_{(C)Sulfate}$ ($\mu M\ yr^{-1}$)‡					1.7-4.4 x 10^{-5}	1.9-4.9 x 10^{-4}
$C_{Sulfate}$ *(μM) accumulated over the last 2.60 billion years*‡					4.4-11.4 x 10^{4}	4.9-12.7 x 10^{5}
$C_{Sulfate}$ *(μM) accumulated over the last 1.1 billion years*‡					1.9-4.9 x 10^{4}	2-5.4 x 10^{5}
Time to produce 97μM sulfate (million years) ‡					2.2-5.6	0.2-0.5
Time to produce 376μM sulfate (million years) ‡					8.4-21.8	0.8-2.0

† The range is defined by two groups of concentration data for U, Th and K: 0.91 ppm, 4.31 ppm and 1.48% [10] to 2 ppm, 9 ppm and 2%[11] and the isotope concentrations in each element: 99.275% ^{238}U in U, 0.721% ^{235}U in U, 100% ^{232}Th in Th, and 0.0117% ^{40}K in K.

↕ Calculated by Equation 2 with W value of 0.0043 and S_i value from 1.5 (for α particle) to 1.14 (for γ particle); see text for data sources.

‡ Considering IROP-derived sulfate only; assuming no sulfate from other sources and not taking into account sulfate loss through sulfate reduction process.

Supplementary Table 3: Calculation of sulfate loss by thermochemical sulfate reduction based on a kinetic isotope model.

Temperature (ºC)	50	100
ε*	-27.6‰	-19.7‰
$\delta^{34}S_{Initial}$[↑]	1.0‰ -5.2‰	
$\delta^{34}S_{Final}$	5.3‰ -8.5‰	
Remaining sulfate fraction (*f*)	0.76-1.0	0.68-0.99
Maximum sulfate loss fraction	0.24	0.32
Maximum sulfate Loss/Remaining ratio	0.31	0.46
Sulfate loss in μM[§]	30-116	45-173

*Data from Kiyosu & Krouse[12].

[↑]Based on the $\delta^{34}S$ range of pyrites (-0.5‰ to 1.8‰)[5] in massive sulfide and the $\delta^{34}S$ increase of 1.5‰ to 3.4‰ in sulfate product by IROP[13].

[§]Calculated from the concentration range (97-376 μM) of the remaining sulfate in the fracture waters (Main text Table 1).

Supplementary Table 4: Calculation of sulfate loss by thermochemical sulfate reduction based on an equilibrium isotope model.

Temperature (ºC)	50	100
α*	0.942	0.954
$\delta^{34}S_{Initial}$[↑]	1.0‰ -5.2‰	
$\delta^{34}S_{Final}$	5.3‰ -8.5‰	
Remaining sulfate fraction (*f*)	0.87-1.0	0.84-1.0
Maximum sulfate loss fraction	0.13	0.16
Maximum sulfate Loss/Remaining ratio	0.15	0.19
Sulfate loss in μM[§]	14-55	19-73

*Calculated from Shanks et al.[14]

[↑]Based on the $\delta^{34}S$ range of pyrites (-0.5‰ to 1.8‰)[5] in massive sulfide and the $\delta^{34}S$ increase of 1.5‰ to 3.4‰ in sulfate product by IROP[13].

[§]Calculated from the concentration range (97-376 μM) of the remaining sulfate in the fracture waters (Main text Table 1).

Supplementary References

1 Hoffman, P. F. Precambrian geology and tectonic history of North America. In *The Geology of North America – An overview* (eds A.W. Bally & A.R. Palmer) 447-512 (The Geological Society of America, 1989).

2 Barrie, C. T. Komatiite flows of the Kidd Creek footwall, Abitibi Subprovince, Canada. In *Economic Geology Monograph 10: The Giant Kidd Creek Volcanogenic Massive Sulfide Deposit, Western Abitibi Subprovince, Canada* (eds M.D. Hannington & C.T. Barrie) 143-162 (the Economic Geology Publishing Co., 1999).

3 Prior, G. J., Gibson, H. L., Watkinson, D. H. & Cook, R. E. Anatomy, lithogeochemistry, and emplacement mechanisms for the QP rhyolite, Kidd Creek Mine, Timmins, Ontario. In *Economic Geology Monograph 10: The Giant Kidd Creek Volcanogenic Massive Sulfide Deposit, Western Abitibi Subprovince, Canada* (eds M.D. Hannington & C.T. Barrie) 123-142 (the Economic Geology Publishing Co., 1999).

4 Bleeker, W. Structure, stratigraphy, and primary setting of the Kidd Creek volcanogenic massive sulfide deposit: A semiquantitative reconstruction. In *Economic Geology Monograph 10: The Giant Kidd Creek Volcanogenic Massive Sulfide Deposit, Western Abitibi Subprovince, Canada* (eds M.D. Hannington & C.T. Barrie) 71-122 (the Economic Geology Publishing Co., 1999).

5 Jamieson, J. W., Wing, B. A., Farquhar, J. & Hannington, M. D. Neoarchean seawater sulfate concentrations from sulfur isotopes in Kidd Creek ore sulfides. *Nature Geoscience* **6**, 61-64 (2013).

6 Kurzweil, F. *et al.* Atmospheric sulfur rearrangement 2.7 billion years ago: Evidence for oxygenic photosynthesis. *Earth Planet. Sci. Lett.* **366**, 17-26 (2013).

7 Hannington, M. D., Barrie, C. T. & Bleeker, W. The giant Kidd Creek volcanogenic massive sulfide deposit, western Abitibi Subprovince, Canada: Preface and introduction. In *Economic Geology Monograph10: The Giant Kidd Creek Volcanogenic Massive Sulfide Deposit, Western Abitibi Subprovince, Canada* (eds M.D. Hannington & C.T. Barrie) 1-30 (the Economic Geology Publishing Co., 1999).

8 Asta, M. P., Gama, J., Soler, J. M., Arvidson, R. S. & Lüttge, A. Interferometric study of pyrite surface reactivity in acidic conditions. *Amer. Miner.* **93**, 508-519 (2008).

9 Hannington, M. D., Bleeker, W., Kjarsgaard, I. Sulfide mineralogy, geochemistry, and ore genesis of the Kidd Creek Deposit: Part I. North, central, and south orebodies. In *Economic Geology Monograph 10: The Giant Kidd Creek Volcanogenic Massive Sulfide Deposit, Western Abitibi Subprovince, Canada* (eds M.D. Hannington & C.T. Barrie) 163-224 (the Economic Geology Publishing Co., 1999).

10 Ketchum, J. W. F., Ayer, J. A., van Breemen, O., Pearson, N. J. & Becker, J. K. Pericontinental crustal growth of the Southwestern Abitibi Subprovince, Canada — U-Pb, Hf, and Nd isotope evidence. *Econ. Geol.* **103**, 1151-1184 (2008).

11 Moulton, B. J. A., Fowler, A. D., Ayer, J. A., Berger, B. R. & Mercier-Langevin, P. Archean subqueous high-silica rhyolite coulées: Examples from the Kidd-Munro Assemblage in the Abitibi Subprovince. *Precam. Res.* **189**, 389-403 (2011).

12 Kiyosu, Y. & Krouse, H. R. The role of organic acid in the abiogenic reduction of sulfate and the sulfur isotope effect. *Geochem. J.* **24**, 21-27 (1990).

13 Lefticariu, L., Pratt, L. M., LaVerne, J. A. & Schimmelmann, A. Anoxic pyrite oxidation by water radiolysis products - A potential source of biosustaining energy. *Earth Planet. Sci. Lett.* **292**, 57-67 (2010).

14 Shanks III, W. C., Bischoff, J. L. & Rosenbauer, R. J. Seawater sulfate reduction and sulfur isotope fractionation in basaltic systems: Interaction of seawater with fayalite and magnetite at 200-350°C. *Geochim. Cosmochim. Acta* **45**, 1977-1995 (1981).
